# Artificial intelligence for the diagnosis of clinically significant prostate cancer based on multimodal data: a multicenter study

**DOI:** 10.1186/s12916-023-02964-x

**Published:** 2023-07-24

**Authors:** Huiyong Zhang, Jin Ji, Zhe Liu, Huiru Lu, Chong Qian, Chunmeng Wei, Shaohua Chen, Wenhao Lu, Chengbang Wang, Huan Xu, Yalong Xu, Xi Chen, Xing He, Zuheng Wang, Xiaodong Zhao, Wen Cheng, Xingfa Chen, Guijian Pang, Guopeng Yu, Yue Gu, Kangxian Jiang, Bin Xu, Junyi Chen, Bin Xu, Xuedong Wei, Ming Chen, Rui Chen, Jiwen Cheng, Fubo Wang

**Affiliations:** 1 Center for Genomic and Personalized Medicine, Guangxi Key Laboratory for Genomic and Personalized Medicine, Guangxi Collaborative Innovation Center for Genomic and Personalized Medicine, Guangxi Medical University, Nanning, 530021 Guangxi China; 2 Department of Urology, Institute of Urology and Nephrology, First Affiliated Hospital of Guangxi Medical University, Nanning, 530021 Guangxi China; 3 Department of Urology, Shanghai Changhai Hospital, Second Military Medical University, Shanghai, 200433 China; 4 Department of Urology, Naval Medical Center, Naval Medical University, Shanghai, 200052 China; 5 Department of Urology, Jinling Hospital, Medical School of Nanjing University, Nanjing, 210002 China; 6 Department of Urology, The First Affiliated Hospital of Xi’an Jiaotong University, Xi’an, 710061 Shaanxi China; 7 Department of Urology, The First People’s Hospital of Yulin, Yulin, 537000 Guangxi China; 8 Department of Urology, Shanghai Ninth People’s Hospital, Shanghai Jiaotong University School of Medicine, Shanghai, 200011 China; 9 Department of Urology, The Second Affiliated Hospital of Fujian Medical University, Quanzhou, 362000 Fujian China; 10 Department of Urology, Zhongda Hospital, Southeast University, Nanjing, China; 11 Department of Urology, The First Affiliated Hospital of Soochow University, Suzhou, 215006 China; 12 Department of Urology, Renji Hospital, Shanghai Jiao Tong University School of Medicine, Shanghai, 200127 China

**Keywords:** Prostate cancer, PCAIDS, Artificial intelligence, Machine learning, Diagnosis

## Abstract

**Background:**

The introduction of multiparameter MRI and novel biomarkers has greatly improved the prediction of clinically significant prostate cancer (csPCa). However, decision-making regarding prostate biopsy and prebiopsy examinations is still difficult. We aimed to establish a quick and economic tool to improve the detection of csPCa based on routinely performed clinical examinations through an automated machine learning platform (AutoML).

**Methods:**

This study included a multicenter retrospective cohort and two prospective cohorts with 4747 cases from 9 hospitals across China. The multimodal data, including demographics, clinical characteristics, laboratory tests, and ultrasound reports, of consecutive participants were retrieved using extract-transform-load tools. AutoML was applied to explore potential data processing patterns and the most suitable algorithm to build the Prostate Cancer Artificial Intelligence Diagnostic System (PCAIDS). The diagnostic performance was determined by the receiver operating characteristic curve (ROC) for discriminating csPCa from insignificant prostate cancer (PCa) and benign disease. The clinical utility was evaluated by decision curve analysis (DCA) and waterfall plots.

**Results:**

The random forest algorithm was applied in the feature selection, and the AutoML algorithm was applied for model establishment. The area under the curve (AUC) value in identifying csPCa was 0.853 in the training cohort, 0.820 in the validation cohort, 0.807 in the Changhai prospective cohort, and 0.850 in the Zhongda prospective cohort. DCA showed that the PCAIDS was superior to PSA or fPSA/tPSA for diagnosing csPCa with a higher net benefit for all threshold probabilities in all cohorts. Setting a fixed sensitivity of 95%, a total of 32.2%, 17.6%, and 26.3% of unnecessary biopsies could be avoided with less than 5% of csPCa missed in the validation cohort, Changhai and Zhongda prospective cohorts, respectively.

**Conclusions:**

The PCAIDS was an effective tool to inform decision-making regarding the need for prostate biopsy and prebiopsy examinations such as mpMRI. Further prospective and international studies are warranted to validate the findings of this study.

**Trial registration:**

Chinese Clinical Trial Registry ChiCTR2100048428. Registered on 06 July 2021.

**Supplementary Information:**

The online version contains supplementary material available at 10.1186/s12916-023-02964-x.

## Background

Prostate cancer (PCa) is a malignancy with the second highest incidence and the fifth highest mortality among tumors affecting males worldwide [1]. The early detection of PCa is particularly imperative for PCa patients. When the tumor spreads beyond the capsule or distant metastasis, therapeutic effectiveness remains limited, and the patient’s prognosis is devastatingly dismal [1–4]. Despite the widespread applications of prostate-specific antigen (PSA), the current detection modality of PCa has yielded huge overdiagnosis, overtreatment of indolent cases, and missing clinically significant cases [5]. Although multiparameter MRI (mp-MRI) has gained great importance in predicting the risk of PCa before biopsy, it is not possible for every man with elevated PSA levels to undergo mpMRI due to the limited recourses of MRI facilities and the high cost of mpMRI. The need for prebiopsy and prempMRI screening and selection is very urgent.

Artificial intelligence (AI) has the potential to revolutionize current clinical practice, such as diagnosis, identification of previously unrecognized images or genomic paradigms associated with disease phenotypes, adjuvant or incorporated singly surgical intervention [6, 7]. As reported, AI systems that integrate electronic case information present outstanding performance in diagnosing lung cancer compared with existing clinical diagnostic criteria and can reduce the missed diagnosis rate of lung cancer by 30.7% [8]. Therefore, AI, with its powerful capacity for information processing, can largely integrate clinical multimodal and multidimensional information resources and is expected to become a revolutionary milestone in the field of early detection and accurate diagnosis for PCa. Here, we established a prostate cancer artificial intelligence diagnostic system (PCAIDS) based on AutoML through processing, modeling, and verification of multimodal data, which might aid in the surveillance and early detection of PCa.

## Methods

### Study population

This is a multicenter, retrospective, diagnostic study that included consecutive clinical patients in nine tertiary medical centers in different regions of China. The study was approved by the local ethics committee (CHEC2021-092), registered in the Chinese Clinical Trial Registry (ChiCTR2100048428), and undertaken according to the Declaration of Helsinki. Informed consent from patients with PCa and controls was acquired in the prospective cohorts. All patients with PCa and controls were confirmed by pathological examination. Histological classification was established according to the WHO classification.

We extracted various features from the subjects, including demographics (height, weight, gender, etc.), imaging reports (abdominal B-ultrasound), and clinical laboratory tests (PSA, routine blood tests, routine urine tests, blood lipids, hepatic function, blood glucose, etc.). Inclusion criteria were (1) the subject scheduled to undergo the initial prostate biopsy; (2) PSA 4–20 ng/mL; (3) ultrasound examinations were completed and associated reports were processed by Natural Language Processing (NLP), with detailed information of upper and lower diameter and left and right diameters; and (4) patients with complete clinical information.

### Study design

**Fig. 1:**
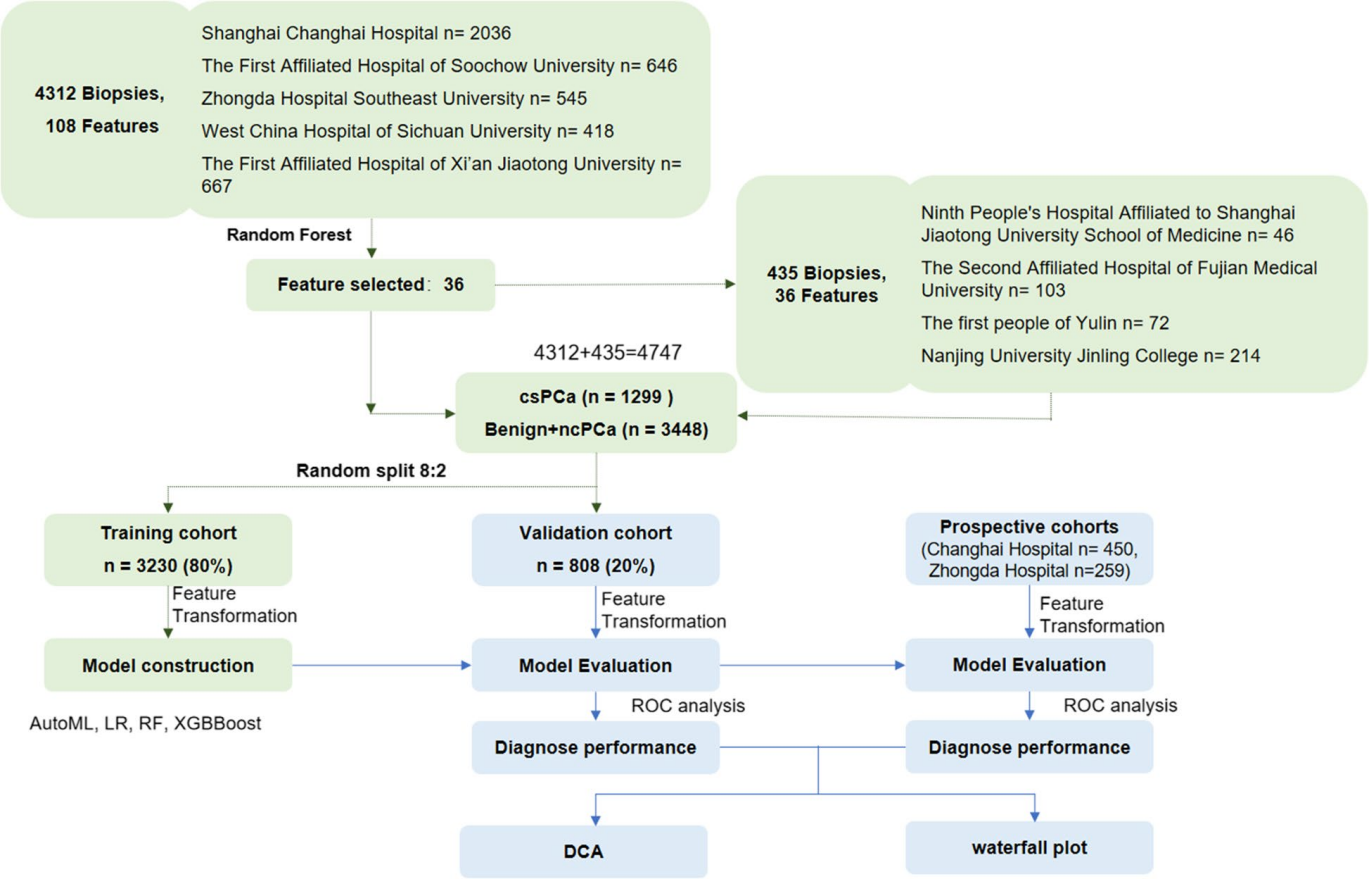
Study design. AutoML, automated machine learning; LR, logistic regression; RF, random forest; ROC, receiver operating characteristic curve; DCA, decision curve analysis

A summary of the workflow and an overview of the cohorts are shown in Fig. 1 according to the statement for transparent reporting of a multivariable prediction model for individual prognosis or diagnosis (TRIPOD) (http://www.equator-network.org/reportingguidelines/tripod-statement/). Specifically, we performed feature selection using data from 4312 biopsy-positive PCa patients and biopsy-negative control patients who underwent prostate biopsy at five clinical sites between May 2008 and December 2019. A total of 435 patients underwent prostate biopsy from January 2016 to December 2021 for subsequent analysis in the other 4 centers.

First, the retrospective data were divided into a training cohort (3230 patients, 80%) and an internal validation cohort (808 cases, 20%). The prospective cohorts included the Shanghai Changhai Hospital prospective cohort (519 cases) and Zhongda Hospital cohort (190 cases). The data of the training cohort were applied to construct models using four algorithms, including AutoML, logistic regression (LR), random forest (RF), and XGBoost. Then, the four models were evaluated, and the classifiers with the best prediction performances were chosen to establish the final diagnostic model. The parameters of the diagnostic model from the training cohort were applied to validate the diagnostic performance of the selected model. The discriminative power of the selected model with PCa was evaluated by the area under the curve (AUC) of the receiver operating characteristic curve (ROC). Then, the clinical value of the model was further evaluated.

### Data preprocessing

*First*, demographics, imaging reports, and clinical laboratory tests of eligible participants were extracted from Shanghai Changhai Hospital, the First Affiliated Hospital of Soochow University, Zhongda Hospital Southeast University, West China Hospital of Sichuan University, and the First Affiliated Hospital of Xi’an Jiaotong University using extract-transform-load (ETL) tools. The NLP module was used to extract the “upper and lower diameters and left and right diameters” reported by ultrasound. Then, all the laboratory tests were subjected to quality control steps for further feature selection. The RF model-based method was used to select candidate features for modeling [9].

Furthermore, we collected the selected features of patients in the Ninth People’s Hospital Affiliated to Shanghai Jiaotong University School of Medicine, the Second Affiliated Hospital of Fujian Medical University, the First People Hospital of Yulin, and Nanjing University Jinling Hospital. All subjects in the abovementioned centers were randomly divided into the training cohort and internal validation cohort at a ratio of 8:2. Patients prospectively collected from Shanghai Changhai Hospital and Zhongda Hospital were used for independent prospective validation.

### Feature transformation

For enumerated or categorical features, the missing values were filled with “NA”; some niche categories, such as “++, +++”, were classified and merged into one category; then, a one-hot encoding method was used to convert to a numeric vector. For continuous indicators, the mean was used to replace missing values, and then the MinMaxScaler method was used for regularization conversion [10]. The conversion formula is ${x}{\prime}=\frac{x-{x}_{min}}{{x}_{max}-{x}_{min}}$, $x^{'}$ is the conversion result, and ${x}_{max}$ and ${x}_{min}$ are the maximum and minimum values of x, respectively.

### AI-based feature selection

The random forest algorithm, a renowned machine learning method, was employed for feature selection. The dataset comprised 4312 biopsy cases, each characterized by 108 different features. This cohort was randomly partitioned into distinct training and validation subsets (8:2) to facilitate robust model training and subsequent performance evaluation. Utilizing the random forest algorithm, the importance of each feature was computed within the training subset. A cumulative contribution threshold of 95% was set to identify the most influential features. Finally, 36 features were selected. The selected features and corresponding contributions are summarized in Additional file 1: Table S1.

### Model training and evaluation

The PCAIDS, an AutoML model, was redeveloped and trained based on Autogluon, one of the AutoML frameworks. We selected three commonly used algorithms in the field of machine learning: logistic regression (LR), random forest (RF), and extreme gradient boosting (XGBoost). Logistic regression is a statistical model used for predicting binary outcomes. Random forest is an ensemble learning method that operates by constructing a multitude of decision trees. Extreme gradient boosting, or XGBoost, is an ensemble tree method that utilizes a gradient boosting framework [11]. We used these algorithms to compare results in the internal validation cohort and two independent prospective cohorts. After data preprocessing and feature transformation, we also compared the predictive performance of PCAIDS with PSA and free PSA/total PSA (fPSA/tPSA). The whole project was implemented in Python 3 (Python 3.7.11) and partly conducted via the packages Scikit-learn (Scikit-learn 0.19.1) and autogluon (0.4.2).

### Statistical analysis

All of the continuous features are presented as medians and interquartile ranges. Missing value cases and categorical variables are presented as numbers and percentages. We used the ROC curve to show the predictive ability of PCAIDS, and we calculated 95% confidence intervals (CIs) for sensitivity and specificity with the bootstrap method [12]. Then, the clinical value was evaluated by decision curve analysis (DCA) and a waterfall plot. We employed the SHAP tool to parse and evaluate the contribution of each predictor [13].

## Results

### Characteristics of the participants

A total of 108 variables from 4747 patients who underwent prostate biopsy were extracted from nine hospitals in China and randomly divided into a training cohort and a validation cohort at a ratio of 8:2. The demographics and clinical characteristics of patients in each center are shown in Additional file 2: Table S2. According to the feature selection results (Additional file 3: Table S3), 36 features were included in the study (Table 1).

**Table 1:** Demographics and clinical characteristics of participants

Parameter	Training cohort			Validation cohort			Changhai prospective cohort			Zhongda prospective cohort		
	Benign+nsPCa	csPCa	*p* value^#^	Benign+nsPCa	csPCa	*p* value^#^	Benign+nsPCa	csPCa	*p* value^#^	Benign+nsPCa	csPCa	*p* value^#^
No. pts (%)	2409 (74.6%)	821 (25.4%)		603 (74.6%)	205 (25.4%)		274 (60.9%)	176 (39.1%)		162 (62.5%)	97 (37.5%)	
Age (media, IQR)	67.00 (61.00–73.00)	70.00 (64.00–76.00)	<0.001	67.00 (61.00–73.00)	72.00 (67.00–76.00)	<0.001	66.00 (61.00–71.00)	69.50 (64.00–75.00)	<0.001	69.00 (63.00–75.00)	70.00 (66.00–76.00)	0.053
PSA (media, IQR)	9.27 (6.78–12.80)	10.80 (7.73–14.51)	<0.001	9.68 (7.12–12.76)	11.64 (8.41–15.29)	<0.001	7.81 (5.91–11.29)	9.50 (6.91–12.88)	0.002	8.06 (5.97–12.06)	9.89 (7.14–12.80)	0.07
B_AREA (media, IQR)	22.79 (17.94–28.60)	17.16 (13.95–21.00)	<0.001	22.95 (17.76–29.69)	16.72 (13.76–20.67)	<0.001	23.04 (18.64–28.56)	17.15 (14.40–23.01)	<0.001	23.77 (19.46–29.68)	17.19 (14.06–20.91)	<0.001
fPSA/PSA (media, IQR)	0.16 (0.11–0.21)	0.11 (0.08–0.16)	<0.001	0.16 (0.11–0.21)	0.11 (0.08–0.15)	<0.001	0.18 (0.13–0.23)	0.13 (0.09–0.17)	<0.001	0.17 (0.13–0.21)	0.12 (0.09–0.15)	<0.001

*nsPCa* Non-significant prostate cancer, *csPCa* Clinical significant prostate cancer, *IQR* Interquartile range, *PSA* Prostate-specific antigen, *tPSA* Total PSA, *B_AREA* Size of B-ultrasound cross-section prostate area

^#^ Mann-Whitney *U* test

### The PCAIDS (AutoML based) showed the highest diagnostic efficacy compared to LR, RF, and XGBoost

**Fig. 2:**
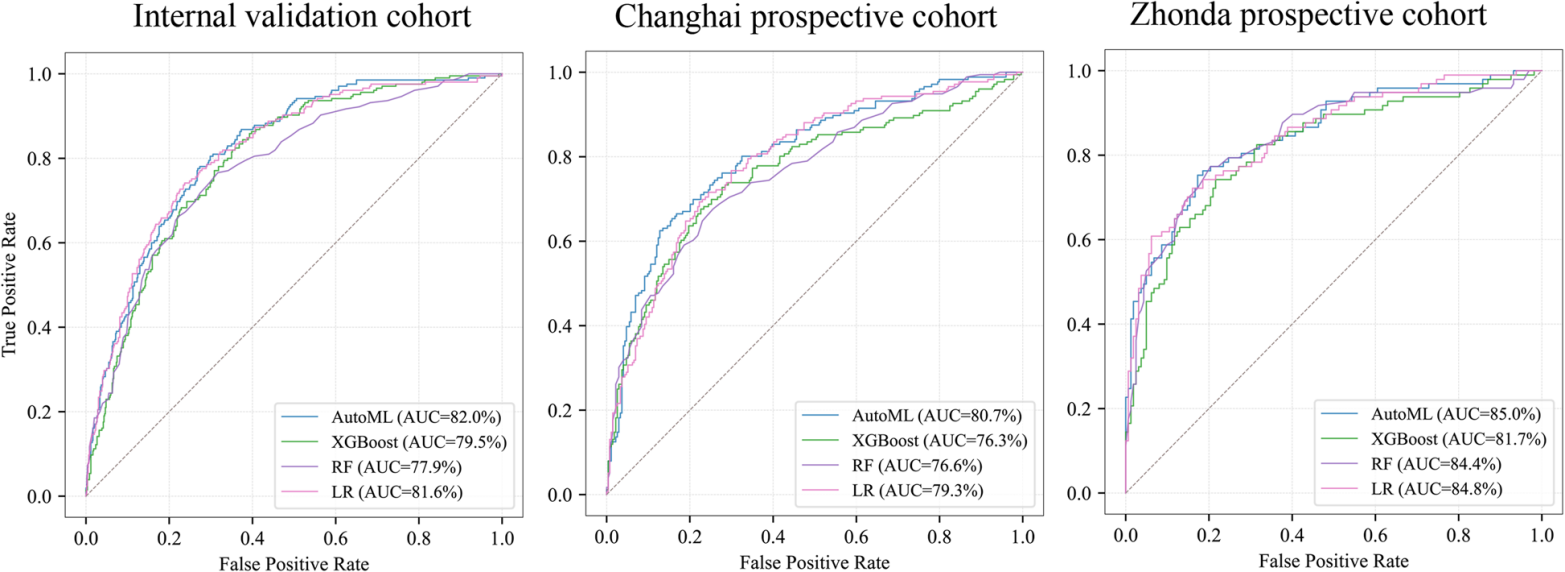
The results of the four algorithms for diagnostic model construction were compared in an internal validation cohort and two prospective cohorts, Chinghai and Zhongdu. AUC, area under the curve; AutoML, automated machine learning; LR, logistic regression; RF, random forest

In the internal validation cohort, our results (Fig. 2, Table 2.) showed that AutoML manifested the highest diagnostic accuracy with an AUC of 0.820 (95% CI: 0.79–0.85) compared to LR of 0.816 (95% CI, 0.78–0.85), RF of 0.779 (95% CI, 0.74–0.82), and XGBoost of 0.795 (95% CI, 0.76–0.83) when distinguishing clinically significant prostate cancer (csPCa) from benign disease and insignificant PCa. Similarly, in two prospective cohorts, AutoML showed an ideal diagnostic performance. In the Changhai prospective cohort, AutoML had the highest AUC of 0.807 (95% CI: 0.76–0.85) versus LR of 0.793 (95% CI: 0.75–0.83), RF of 0.766 (95% CI: 0.72–0.81) and XGBoost of 0.763 (95% CI: 0.71–0.81). In the Zhongda prospective cohort, AutoML also had the highest AUC of 0.850 (95% CI: 0.80–0.89) versus LR of 0.848 (95% CI: 0.80–0.90), RF 0.844 (95% CI: 0.79–0.90) and XGBoost of 0.817 (95% CI: 0.76–0.87). Based upon the above results, AutoML was used to construct the final model.

**Table 2:** AUC results using 4 algorithms, PSA and fPSA/fPSA in the validation cohort

AUC(95%CI)	Validation cohort	Changhai cohort	Zhongda cohort
LR	0.816 (0.78–0.85)	0.793 (0.75–0.83)	0.848 (0.80–0.90)
RF	0.779 (0.74–0.81)	0.766 (0.72–0.81)	0.844 (0.79–0.90)
XGBoost	0.795 (0.76–0.83)	0.763 (0.71–0.81)	0.817 (0.76–0.87)
AutoML	0.820 (0.79–0.85)	0.807 (0.76–0.85)	0.850 (0.80–0.89)
PSA	0.616 (0.57–0.66)	0.593 (0.54–0.65)	0.583 (0.51–0.65)
fPSA/PSA	0.675 (0.63–0.72)	0.675 (0.62–0.73)	0.738 (0.67–0.80)

*AUC* Area under receiver operating characteristic, *AutoML* Automated machine learning, *LR* Logistic regression, *RF* Random forest

### Diagnostic efficacy of the PCAIDS

**Fig. 3:**
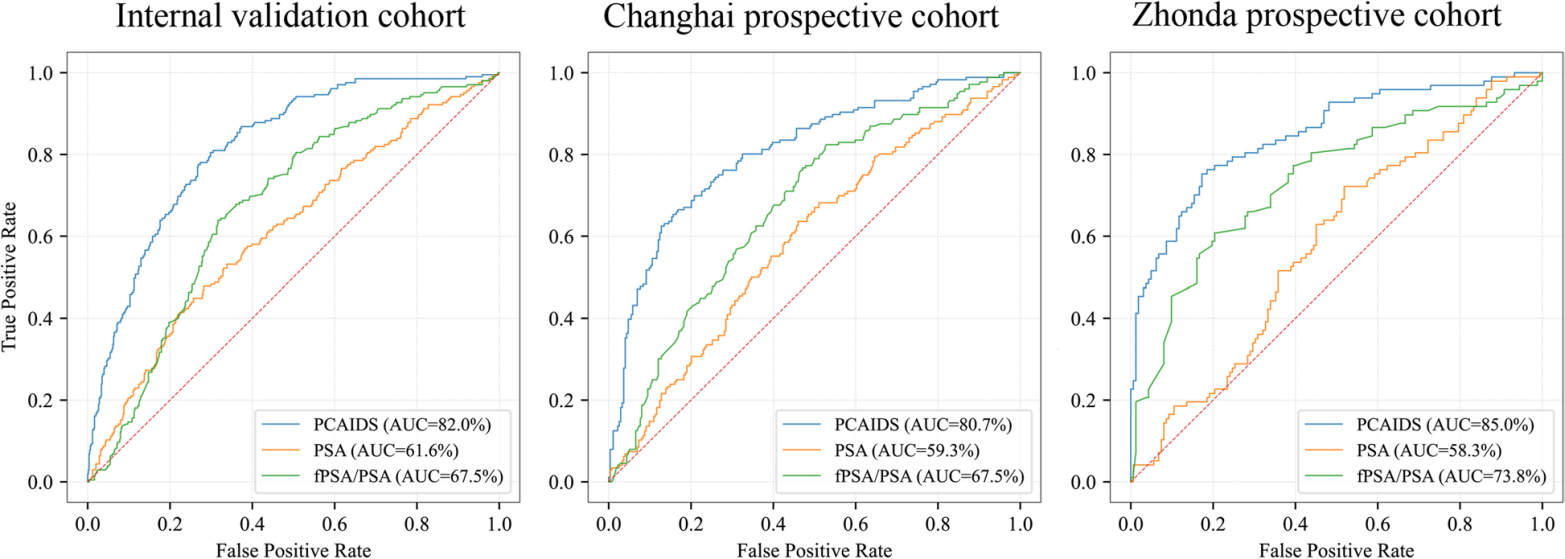
The diagnostic performance AUC of AutoML outperformed PSA, fPSA/tPSA, and SOC in an internal validation cohort and two prospective cohorts, Chinghai and Zhongdu. AUC, area under the curve; AutoML, automated machine learning; PSA, prostate cancer-specific antigen; SOC, standard of care

The PCAIDS had a higher diagnostic efficacy than PSA and fPSA/tPSA, with AUCs of 0.820 (95% CI: 0.79–0.85), 0.616 (95% CI: 0.57–0.66) and 0.675 (95% CI: 0.63–0.71) in distinguishing csPCa, respectively (Fig. 3, Table 2). In the Changhai prospective cohort and the Zhongda prospective cohort, the PCAIDS was superior to PSA and fPSA/tPSA, with higher AUC values (Fig. 3).

### The clinical benefits of the PCAIDS

**Fig. 4:**
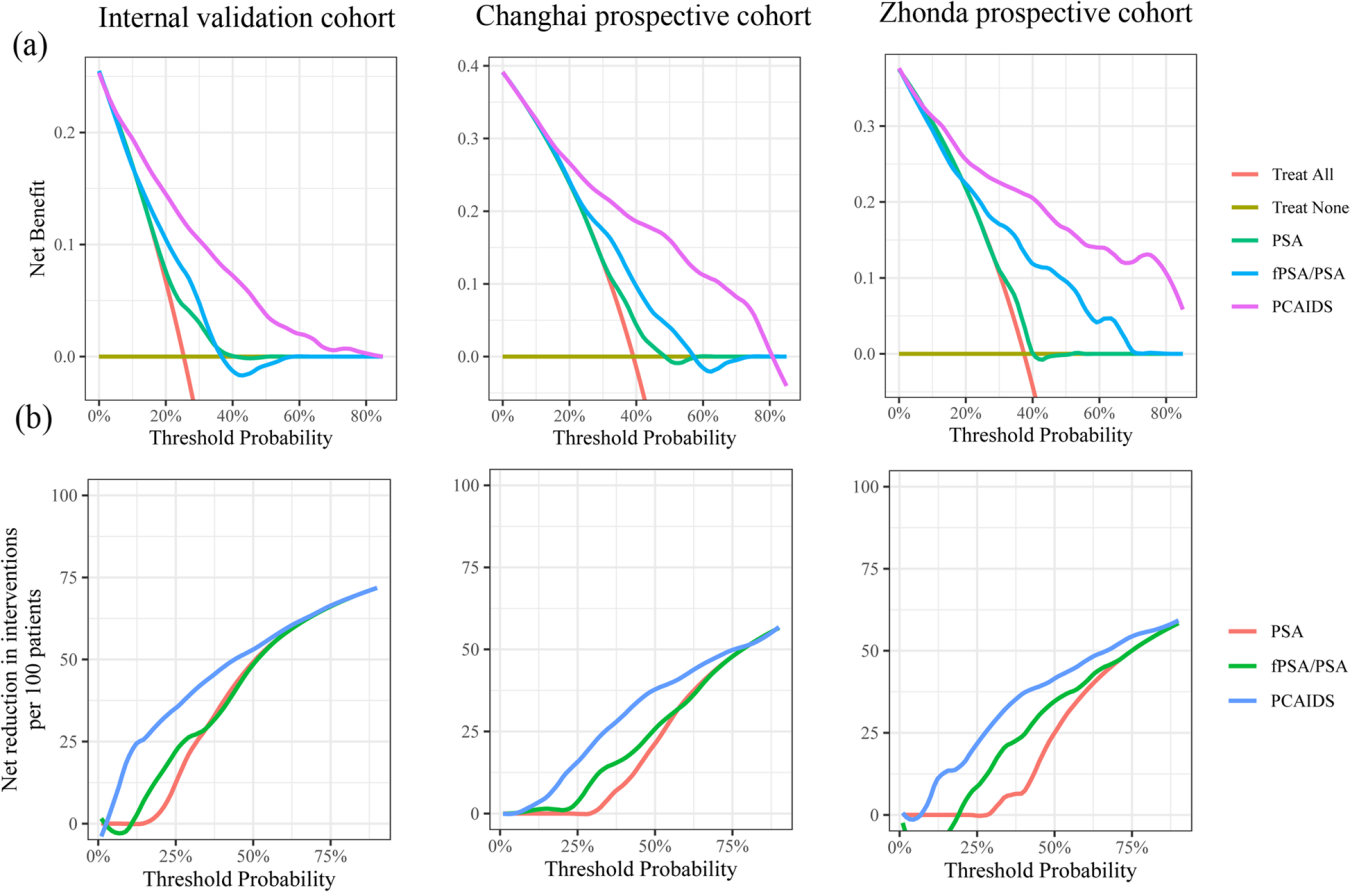
DCA for AutoML, PSA, fPSA/tPSA, and SOC in an internal validation cohort and two prospective cohorts, Chinghai and Zhongdu. **a** DCA shows that AutoML presented the highest net benefit across all threshold probabilities for PCa. The horizontal gray‒green lines parallel to the *x*-axis represent no patient undergoing a biopsy (Treat None). The red line indicates that all the patients will have PCa (Treat All). **b** AutoML outperformed PSA, fPSA/tPSA, and SOC in net reduction per 100 patient interventions at all thresholds. DCA, decision curve analysis; AutoML, automated machine learning; PSA, prostate cancer-specific antigen; fPSA/tPSA, free PSA/total PSA; SOC, standard of care

We also investigated the net clinical value of the PCAIDS using DCA by comparing PSA and fPSA/PSA over a range of probabilities. In this analysis, the PCAIDS had the highest net benefit in the validation cohort, and the Changhai prospective cohort and Zhongda prospective cohort demonstrated significant clinical utility when compared with PSA and fPSA/PSA (Fig. 4).

**Fig. 5:**
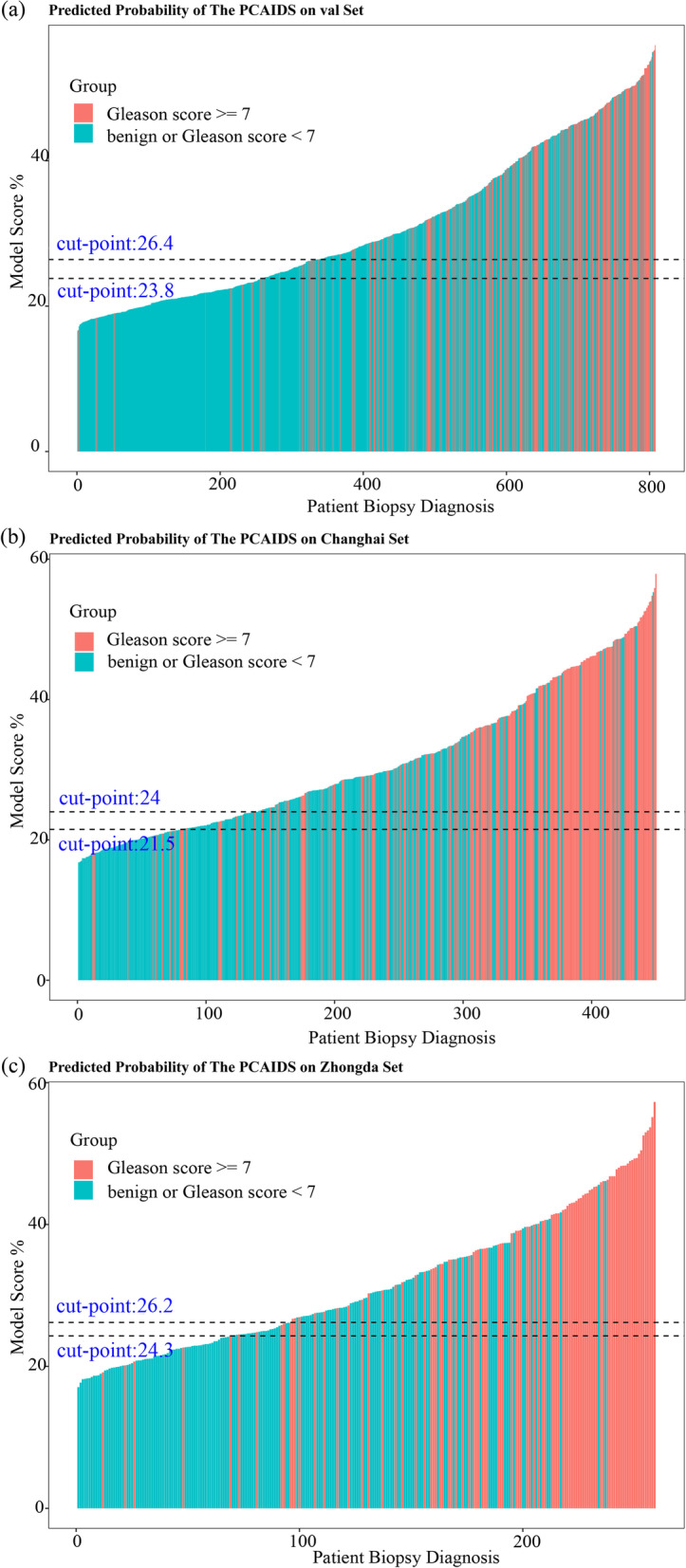
Waterfall plot of AutoML related to prostate biopsy results in an internal validation cohort and two prospective cohorts, Chinghai and Zhongdu. Each bar represents an individual. Red indicates ISUP grade ≥ two tumors (Gleason score ≥ 7); blue indicates ISUP grade of one tumor (Gleason score < 7). Two black horizontal lines represent the cutoff points of 26.4 at a sensitivity of 90% and 23.8 at a sensitivity of 95%. AutoML, automated machine learning; ISUP, International Society of Urological Pathology

In the validation cohort, the distribution of biopsy results was depicted in a waterfall plot (Fig. 5). The cutoff values were set according to the sensitivity of 90% and 95%. When the cutoff value with 95% sensitivity was applied (23.8%), the PCAIDS indicated a negative predictive value (NPV) of 96.15% and PPV of 35.58%, preventing 32.18% of unnecessary biopsies at risk and missing only 4.88% of cases of csPCa (Table 3).

**Table 3:** Performance of AutoML in the validation cohort, Changhai prospective cohorts, and Zhongda prospective cohorts at 95% sensitivity

Cohort	Biopsy result		Total	Performance, %
	csPCa	Benign+nsPCa		
Validation internal cohort, cut-off value= 23.8%				
AutoML probability > cut point	195	353	548	Sensitivity= 95.1
AutoML probability <= cut point	10	250	260	Specificity= 41.5
Total	205	603	808	PPV= 35.6, NPV= 96.2
csPCa biopsy prevalence %	25.4	Fraction predicted negative	32.2	Missing= 4.9
Changhai prospective cohort, cut-off value= 21.5%				
AutoML probability > cut point	168	203	371	Sensitivity= 95.5
AutoML probability <= cut point	8	71	79	Specificity= 25.9
Total	176	274	450	PPV=45.3, NPV=89.9
csPCa biopsy prevalence %	39.1	Fraction predicted negative	17.6	Missing=4.5
Zhongda prospective cohort, cut-off value= 24.3%				
AutoML probability > cut point	93	98	191	Sensitivity=95.9
AutoML probability <= cut point	4	64	68	Specificity=39.5
Total	97	162	259	PPV=48.7, NPV=94.1
csPCa biopsy prevalence %	37.5	Fraction predicted negative	26.3	Missing=4.1

*nsPCa* Non-significant prostate cancer, *csPCa* Clinically significant prostate cancer, *AutoML* Automated machine learning, *LR* Logistic regression, *NLR* Negative likelihood ratio, *NPV* Negative predictive value, *PLR* Positive likelihood ratio, *PPV* Positive predictive value

In the Changhai prospective cohort, when the cutoff value with 95% sensitivity was applied (21.5%), the PCAIDS showed an NPV of 89.9% and PPV of 45.3%, preventing 17.6% of unnecessary biopsies and missing 4.5% of csPCa cases. In the Zhongda prospective cohort, the PCAIDS showed an NPV of 94.1% and PPV of 48.7% at the cutoff value of 95% sensitivity (24.3%), contributing to the reduction of 26.3% unnecessary biopsies with 4.1% csPCa missed.

## Discussion

We proposed the PCAIDS, an AutoML-based model, for the prediction of csPCa based on quick and economic routinely performed clinical examinations. The PCAIDS incorporated multimodal and multidimensional data, including laboratory tests, imaging tests, and demographic data, revealing encouraging discriminative power with AUCs of 0.820 in the validation cohort and 0.807 and 0.850 in the two prospective test cohorts.

Compared with previous prediction models, such as the ERSPC-RC [14], PCPT-RC [15] and CPCC-RC [16], the PCAIDS, for the first time, evaluated over 100 multimodal features with AI-based algorithms. These features, including demographics, laboratory tests, and imaging examinations, were assessed by a series of AI algorithms. Among these AI algorithms, AutoML outperformed logistic regression, random forest, and XGBoost. AutoML has become a popular and efficient modeling tool for data science that uses k-fold cross-validation through varying optimization algorithms, such as grid search, random search, and genetic algorithm (GA), to scan different feature combinations, feature transformations, supervised algorithms, and their corresponding hyperparameter combinations implemented in AutoWEKA [17], Autogluon [18], AutoSklearn 2.0 [19], and TPOT, [20] thereby identifying the optimal machine learning pipeline.

Additionally, AI-based methods have the potential to analyze high-volume data and to discover nonlinear and interactive prediction information. For cancer diagnosis, there were huge possibilities that currently applied predictive models only included a proportion of effective predictors. Although the application of AI-based methods may not always outperform linear models, the advantage of involving more features could help the models to be more stable and more applicable for different populations.

In this aspect, Jungyo Suh et al. proposed the possibility of applying AI-based algorithms in the prediction of prostate biopsy. They developed an AI-based prediction tool with PSA, total prostate volume, age, hypoechoic lesion on ultrasonography, transitional zone volume, testosterone, and fPSA [21]. This study showed the promising future of using AI-based algorithms in predicting PCa; however, the investigated features were of limited number. To some extent, AI-based algorithms were not ideal for the analysis of limited features, which could have been done by traditional methods. In predicting colon cancer, researchers applied AI-based methods to data from health maintenance organizations by evaluating analytes from standard laboratory records, including hematology, liver function, and metabolism [22]. In breast cancer, the notion of applying AI-based methods to diagnose breast cancer was validated, and age, body mass index (BMI), glucose, insulin, homeostasis model assessment (HOMA), leptin, adiponectin, resistin and chemokine monocyte chemoattractant protein 1 (MCP1) attributes were used in the prediction model [23]. Further studies validated that routine blood analysis features had a boosted performance for breast cancer diagnosis and supported the notion that this approach is of great potential to be used in a widespread manner to detect cancers [24]. These studies suggested the possibility of using routine health examinations to predict cancer based on AI algorithms.

The clinical scenario for the application of PCAIDS is between PSA-based screening and novel tests predicting PCa, including mpMRI, urinary PCA3 test, 4kScore, and Prostate Health Index. MpMRI, a potent modality in predicting biopsy results, is of great importance in patients who are at high risk of PCa. However, the application of mpMRI is limited by the accessibility of MRI machines and the professionalism of the radiologists who interpret the images. Meanwhile, these biomarkers were only available for patients in some countries and regions. In addition, mpMRI and these novel biomarkers are associated with high costs in most countries. The application of PCAIDS, on the other hand, does not require special examination equipment. The features included in the model were common, routinely performed, quick, and economic tests, which were also needed for a general health check-up. The application of B-ultrasound in evaluating the size of the prostate is also accessible for almost every hospital. In general, this AI-based modality is not here to perfect the diagnostic modality with mpMRI and novel biomarkers, rather than replacing them.

AutoML has the flaw of interpretability, which is consistently met with skepticism, similar to other complex models, especially in the medical field. To this end, we applied the SHAP [13] tool to explore the contribution of individual features to the model. To explore the rationality of this contribution, we also examined the interpretability of the LR compared to SHAP (Additional file 4: Figure S1). First, the contribution of the key variables (the cross-sectional area of the prostate (B_AREA), AGE, and fPSA) is basically the same in the two prediction modalities. This is similar to the previous conclusions obtained by the RF model (Additional file 1: Table S1). Second, the SHAP value from AutoML is roughly the same as the importance of LR calculated by model coefficients. Third, B_AREA is the most important variable. Significantly, the risk of PCa did not increase with B_AREA, which may be due to the increased concertation of PSA produced by a larger prostate, misstating that the risk of PCa and fPSA/tPSA are similar. In addition, age played the second most important role in the prediction model. Thereafter, the risk of PCa increases with age, which is intuitive, and the same holds true for other clinical indicators, although no direct cause can be inferred.

One of the limitations of this study is the lack of a head-to-head comparison with mpMRI or other novel biomarkers. However, the clinical scenario of this prediction mode is not to replace novel diagnostic methods but to assist in decision-making for novel diagnostic methods. In addition, we introduced the dimensions of the prostate from the B ultrasound in the model, and there might be inter- and intrarater differences among different centers in terms of ultrasound results. Furthermore, ultrasound images were not included in this study due to the lack of image storage in all centers. We believe that future studies may incorporate the images captured during ultrasound examinations. The findings of this study are applicable primarily to Asian populations due to the vast discrepancy between Asian and Caucasian patients. In the future, we intend to collect data from various populations to adapt our model to different ethnic groups. Finally, the performance of the PCAIDS is not better than that of the other algorithms, including LR. However, it is important to note that in the study, given the serious implications of missing a prostate cancer diagnosis, prioritizing sensitivity rather than specificity was chosen. This decision was made understanding that it might increase the false positive rate, but it's a reasonable trade-off given the potential severity of a missed diagnosis, where high sensitivity can often lead to lower specificity. We consider that further validation studies may help us to show its wide applicability.

## Conclusion

The AutoML-based PCAIDS was a real-time, noninvasive, easy-to-use tool that could be applied for the prediction of csPCa in multimodal routine clinical examinations. The system presented greater diagnostic performance and clinical utility in detecting csPCa than traditional predictors. The PCAIDS was an effective tool for physicians to decide the need for prostate biopsy and prebiopsy examinations such as mpMRI. Further perspective and international studies are warranted to validate the findings of this study.

## Supplementary Information

**Additional file 1: Table S1.** Selected featuresin the Prostate Cancer Artificial Intelligence Diagnostic System.

**Additional file 2: Table S2.** Demographics andclinical characteristics of participants.

**Additional file 3: Table S3.** Clinical researchcenters involved in this study.

**Additional file 4: Figure S1.** Featureimportance analysis uses AutoML. (a) SHAP summary plot. SHAP feature importancemeasured as the mean absolute Shapley values. (b).

## Data Availability

The relevant data have been presented in the article. Correspondence and requests for materials should be addressed to F.W. and R.C.

## Abbreviations

AI: Artificial intelligence
AUC: Area under the curve
AutoML: Automated machine learning
csPCa: Clinically significant prostate cancer
DCA: Decision curve analysis
ETL: Extract-transform-load
fPSA/tPSA: Free PSA/total PSA
GA: Genetic Algorithm
LR: Logistic regression
mp-MRI: Multiparameter MRI
NLP: Natural Language Processing
PCa: Prostate cancer
PCAIDS: Prostate Cancer Artificial Intelligence Diagnostic System
PSA: Prostate-specific antigen
RF: Random forest
ROC: Receiver operating characteristic
TRIPOD: Transparent reporting of a multivariable prediction model for individual prognosis or diagnosis
XGBoost: Extreme gradient boosting
