# Supplementary material for: Artificial intelligence for the diagnosis of clinically significant prostate cancer based on multimodal data: a multicenter study
Source: BMC Med. 2023 Jul 24;21:270. doi: 10.1186/s12916-023-02964-x (PMC10367399; doi:10.1186/s12916-023-02964-x)
Supplement: Supplementary file 1 — Additional file 1: Table S1. Selected featuresin the Prostate Cancer Artificial Intelligence Diagnostic System. [file 12916_2023_2964_MOESM1_ESM.docx]

**Additional file 1: Table S1** Selected features in the Prostate Cancer Artificial Intelligence Diagnostic System.

| **number** | **Feature** | **abbreviation** | **Importance** |
| --- | --- | --- | --- |
| 1 | The cross-sectional area of the prostate | B_AREA | 0.117637 |
| 2 | The ratio of FPSA to PSA | fPSA/PSA | 0.055589 |
| 3 | Age | AGE | 0.0519 |
| 4 | Prostate protests specific | PSA | 0.043084 |
| 5 | Body mass index | BMI | 0.035226 |
| 6 | The ratio of PLT to LYMPH | PLR | 0.034453 |
| 7 | Free PSA | fPSA | 0.032971 |
| 8 | The ratio of NEUT to LYMPH | NLR | 0.03225 |
| 9 | Neutrophil count | NEUT | 0.03051 |
| 10 | The ratio of NEUT to NMT | NMR | 0.026019 |
| 11 | Blood cholesterol | Glu | 0.025309 |
| 12 | Hemoglobin | HGB | 0.024645 |
| 13 | Urine specific gravity | SG | 0.024426 |
| 14 | Platelet count | PLT | 0.024207 |
| 15 | Uric acid | UA | 0.02389 |
| 16 | Blood urea nitrogen | BUN | 0.023192 |
| 17 | Lymphocyte count | LY | 0.023183 |
| 18 | Sodium | EOS | 0.022885 |
| 19 | Lymphocyte percentage | LYMPH | 0.022836 |
| 20 | Total bilirubin | Tbil | 0.021978 |
| 21 | Creatinine | Cr | 0.021762 |
| 22 | Potassium | K | 0.021344 |
| 23 | Total protein | TP | 0.021298 |
| 24 | Direct bilirubin | DBIL | 0.020784 |
| 25 | Alkaline phosphatase | ALP | 0.02062 |
| 26 | Albumin | Alb | 0.020472 |
| 27 | Calcification or calculus of the prostate | Calc_Prost | 0.020087 |
| 28 | Glutamyl transpeptidase | GGT | 0.019904 |
| 29 | Sodium | Na | 0.019861 |
| 30 | Indirect bilirubin | IBIL | 0.01967 |
| 31 | Red blood cell count | RBC | 0.017639 |
| 32 | Red cell distribution width | RDW | 0.017547 |
| 33 | Mean corpuscular hemoglobin concentration | MCHC | 0.017537 |
| 34 | Mean corpuscular volume | MCV | 0.017008 |
| 35 | White blood cell count | WBC | 0.015516 |
| 36 | Monocyte count | NMT | 0.012761 |
