# Supplementary material for: Artificial intelligence for the diagnosis of clinically significant prostate cancer based on multimodal data: a multicenter study
Source: BMC Med. 2023 Jul 24;21:270. doi: 10.1186/s12916-023-02964-x (PMC10367399; doi:10.1186/s12916-023-02964-x)
Supplement: Supplementary file 2 — Additional file 2: Table S2. Demographics andclinical characteristics of participants. [file 12916_2023_2964_MOESM2_ESM.docx]

**Additional file 2: Table S2.** Demographics and clinical characteristics of participants.

| **Parameter** | | **Training Cohort** | | | **Validation Cohort** | | | **Changhai prospective cohort** | | | **Zhongda prospective cohort** | | |
| --- | --- | --- | --- | --- | --- | --- | --- | --- | --- | --- | --- | --- | --- |
|  |  | **Benign+nsPCa** | **csPCa** | **p value** | **Benign+nsPCa** | **csPCa** | **p value** | **Benign+nsPCa** | **csPCa** | **p value** | **Benign+nsPCa** | **csPCa** | **p value** |
| WBC | |  |  | 0.009 |  |  | 0.721 |  |  | 0.253 |  |  | 0.000 |
|  | No. pts (%) | 2409(74.6%) | 821(25.4%) |  | 603(74.6%) | 205(25.4%) |  | 274(60.9%) | 176(39.1%) |  | 162(62.5%) | 97(37.5%) |  |
|  | Media | 0.94 | 0.97 |  | 0.94 | 0.91 |  | 0.88 | 0.83 |  | 0.99 | 0.91 |  |
|  | IQR | 0.78-1.20 | 0.79-1.22 |  | 0.78-1.21 | 0.76-1.20 |  | 0.73-1.03 | 0.70-0.99 |  | 0.83-1.17 | 0.76-1.02 |  |
|  | Missing | 307(9.5%) | 61(1.9%) |  | 74(9.2%) | 13(1.6%) |  | 7(1.6%) | 6(1.3%) |  | 1(0.4%) | 1(0.4%) |  |
| RBC | |  |  | 0.002 |  |  | 0.822 |  |  | 0.212 |  |  | 0.000 |
|  | No. pts (%) | 2409(74.6%) | 821(25.4%) |  | 603(74.6%) | 205(25.4%) |  | 274(60.9%) | 176(39.1%) |  | 162(62.5%) | 97(37.5%) |  |
|  | Media | 0.97 | 0.98 |  | 0.98 | 0.97 |  | 1.00 | 0.99 |  | 0.89 | 0.97 |  |
|  | IQR | 0.90-1.06 | 0.90-1.07 |  | 0.90-1.05 | 0.90-1.04 |  | 0.94-1.07 | 0.92-1.05 |  | 0.82-0.96 | 0.91-1.04 |  |
|  | Missing | 303(9.4%) | 60(1.9%) |  | 74(9.2%) | 13(1.6%) |  | 7(1.6%) | 6(1.3%) |  | 1(0.4%) | 1(0.4%) |  |
| HGB | |  |  | 0.006 |  |  | 0.667 |  |  | 0.914 |  |  | 0.000 |
|  | No. pts (%) | 2409(74.6%) | 821(25.4%) |  | 603(74.6%) | 205(25.4%) |  | 274(60.9%) | 176(39.1%) |  | 162(62.5%) | 97(37.5%) |  |
|  | Media | 1.00 | 1.01 |  | 1.00 | 1.02 |  | 1.04 | 1.04 |  | 0.90 | 1.02 |  |
|  | IQR | 0.93-1.09 | 0.93-1.11 |  | 0.92-1.09 | 0.92-1.09 |  | 0.97-1.10 | 0.96-1.10 |  | 0.83-0.96 | 0.96-1.09 |  |
|  | Missing | 302(9.3%) | 60(1.9%) |  | 74(9.2%) | 13(1.6%) |  | 8(1.8%) | 6(1.3%) |  | 1(0.4%) | 1(0.4%) |  |
| MCV | |  |  | 0.001 |  |  | 0.775 |  |  | 0.276 |  |  | 0.004 |
|  | No. pts (%) | 2409(74.6%) | 821(25.4%) |  | 603(74.6%) | 205(25.4%) |  | 274(60.9%) | 176(39.1%) |  | 162(62.5%) | 97(37.5%) |  |
|  | Media | 1.02 | 1.02 |  | 1.01 | 1.02 |  | 1.01 | 1.02 |  | 0.99 | 1.01 |  |
|  | IQR | 0.99-1.06 | 0.99-1.07 |  | 0.98-1.05 | 0.99-1.06 |  | 0.99-1.04 | 0.99-1.05 |  | 0.96-1.02 | 0.99-1.04 |  |
|  | Missing | 302(9.3%) | 60(1.9%) |  | 74(9.2%) | 13(1.6%) |  | 7(1.6%) | 6(1.3%) |  | 1(0.4%) | 1(0.4%) |  |
| MCHC | |  |  | 0.001 |  |  | 0.639 |  |  | 0.132 |  |  | 0.002 |
|  | No. pts (%) | 2409(74.6%) | 821(25.4%) |  | 603(74.6%) | 205(25.4%) |  | 274(60.9%) | 176(39.1%) |  | 162(62.5%) | 97(37.5%) |  |
|  | Media | 1.01 | 1.02 |  | 1.02 | 1.02 |  | 1.02 | 1.02 |  | 1.01 | 1.02 |  |
|  | IQR | 0.99-1.04 | 0.99-1.05 |  | 0.99-1.04 | 0.99-1.04 |  | 1.00-1.04 | 1.00-1.04 |  | 0.99-1.02 | 1.00-1.04 |  |
|  | Missing | 302(9.3%) | 60(1.9%) |  | 74(9.2%) | 13(1.6%) |  | 7(1.6%) | 6(1.3%) |  | 1(0.4%) | 1(0.4%) |  |
| RDW | |  |  | 0.000 |  |  | 0.807 |  |  | 0.804 |  |  | 0.000 |
|  | No. pts (%) | 2409(74.6%) | 821(25.4%) |  | 603(74.6%) | 205(25.4%) |  | 274(60.9%) | 176(39.1%) |  | 162(62.5%) | 97(37.5%) |  |
|  | Media | 0.99 | 0.99 |  | 0.99 | 0.99 |  | 0.95 | 0.95 |  | 1.00 | 0.96 |  |
|  | IQR | 0.95-1.05 | 0.95-1.06 |  | 0.95-1.05 | 0.95-1.05 |  | 0.92-0.99 | 0.92-0.98 |  | 0.96-1.05 | 0.93-1.00 |  |
|  | Missing | 302(9.3%) | 60(1.9%) |  | 74(9.2%) | 13(1.6%) |  | 7(1.6%) | 6(1.3%) |  | 2(0.8%) | 1(0.4%) |  |
| PLT | |  |  | 0.019 |  |  | 0.953 |  |  | 0.046 |  |  | 0.006 |
|  | No. pts (%) | 2409(74.6%) | 821(25.4%) |  | 603(74.6%) | 205(25.4%) |  | 274(60.9%) | 176(39.1%) |  | 162(62.5%) | 97(37.5%) |  |
|  | Media | 0.93 | 0.94 |  | 0.92 | 0.90 |  | 0.98 | 0.96 |  | 0.83 | 0.91 |  |
|  | IQR | 0.75-1.17 | 0.75-1.19 |  | 0.76-1.19 | 0.74-1.13 |  | 0.83-1.18 | 0.81-1.14 |  | 0.68-1.01 | 0.74-1.09 |  |
|  | Missing | 302(9.3%) | 60(1.9%) |  | 74(9.2%) | 13(1.6%) |  | 7(1.6%) | 6(1.3%) |  | 1(0.4%) | 1(0.4%) |  |
| LYMPH | |  |  | 0.000 |  |  | 0.918 |  |  | 0.030 |  |  | 0.000 |
|  | No. pts (%) | 2409(74.6%) | 821(25.4%) |  | 603(74.6%) | 205(25.4%) |  | 274(60.9%) | 176(39.1%) |  | 162(62.5%) | 97(37.5%) |  |
|  | Media | 0.84 | 0.89 |  | 0.83 | 0.80 |  | 0.95 | 1.00 |  | 0.71 | 0.85 |  |
|  | IQR | 0.65-1.06 | 0.69-1.16 |  | 0.64-1.07 | 0.61-1.01 |  | 0.71-1.13 | 0.82-1.15 |  | 0.51-0.88 | 0.68-1.10 |  |
|  | Missing | 302(9.3%) | 60(1.9%) |  | 74(9.2%) | 13(1.6%) |  | 9(2.0%) | 6(1.3%) |  | 1(0.4%) | 1(0.4%) |  |
| LY | |  |  | 0.005 |  |  | 0.157 |  |  | 0.042 |  |  | 0.266 |
|  | No. pts (%) | 2409(74.6%) | 821(25.4%) |  | 603(74.6%) | 205(25.4%) |  | 274(60.9%) | 176(39.1%) |  | 162(62.5%) | 97(37.5%) |  |
|  | Media | 0.71 | 0.74 |  | 0.71 | 0.65 |  | 0.70 | 0.70 |  | 0.68 | 0.71 |  |
|  | IQR | 0.54-0.94 | 0.59-1.02 |  | 0.53-0.93 | 0.51-0.92 |  | 0.57-0.88 | 0.57-0.87 |  | 0.55-0.88 | 0.54-0.86 |  |
|  | Missing | 302(9.3%) | 60(1.9%) |  | 74(9.2%) | 13(1.6%) |  | 7(1.6%) | 6(1.3%) |  | 1(0.4%) | 1(0.4%) |  |
| NEUT | |  |  | 0.912 |  |  | 0.815 |  |  | 0.271 |  |  | 0.826 |
|  | No. pts (%) | 2409(74.6%) | 821(25.4%) |  | 603(74.6%) | 205(25.4%) |  | 274(60.9%) | 176(39.1%) |  | 162(62.5%) | 97(37.5%) |  |
|  | Media | 0.96 | 0.97 |  | 0.97 | 0.95 |  | 0.83 | 0.73 |  | 0.99 | 0.90 |  |
|  | IQR | 0.74-1.35 | 0.75-1.33 |  | 0.76-1.36 | 0.76-1.29 |  | 0.64-1.05 | 0.62-0.92 |  | 0.81-1.35 | 0.67-1.11 |  |
|  | Missing | 302(9.3%) | 60(1.9%) |  | 74(9.2%) | 13(1.6%) |  | 7(1.6%) | 6(1.3%) |  | 1(0.4%) | 1(0.4%) |  |
| NMT | |  |  | 0.439 |  |  | 0.812 |  |  | 0.428 |  |  | 0.897 |
|  | No. pts (%) | 2409(74.6%) | 821(25.4%) |  | 603(74.6%) | 205(25.4%) |  | 274(60.9%) | 176(39.1%) |  | 162(62.5%) | 97(37.5%) |  |
|  | Media | 0.91 | 0.93 |  | 0.91 | 0.91 |  | 1.04 | 1.04 |  | 1.11 | 1.03 |  |
|  | IQR | 0.66-1.20 | 0.65-1.26 |  | 0.67-1.20 | 0.72-1.18 |  | 0.80-1.30 | 0.87-1.28 |  | 0.89-1.46 | 0.85-1.22 |  |
|  | Missing | 303(9.4%) | 60(1.9%) |  | 75(9.3%) | 13(1.6%) |  | 7(1.6%) | 6(1.3%) |  | 1(0.4%) | 1(0.4%) |  |
| EOS | |  |  | 0.076 |  |  | 0.673 |  |  | 0.056 |  |  | 0.070 |
|  | No. pts (%) | 2409(74.6%) | 821(25.4%) |  | 603(74.6%) | 205(25.4%) |  | 274(60.9%) | 176(39.1%) |  | 162(62.5%) | 97(37.5%) |  |
|  | Media | 0.60 | 0.60 |  | 0.60 | 0.60 |  | 0.60 | 0.60 |  | 0.67 | 0.60 |  |
|  | IQR | 0.33-1.00 | 0.33-1.00 |  | 0.25-0.80 | 0.27-1.00 |  | 0.40-1.00 | 0.40-0.80 |  | 0.33-1.00 | 0.40-1.00 |  |
|  | Missing | 302(9.3%) | 60(1.9%) |  | 74(9.2%) | 14(1.7%) |  | 7(1.6%) | 6(1.3%) |  | 1(0.4%) | 1(0.4%) |  |
| Glu | |  |  | 0.012 |  |  | 0.883 |  |  | 0.484 |  |  | 0.512 |
|  | No. pts (%) | 2409(74.6%) | 821(25.4%) |  | 603(74.6%) | 205(25.4%) |  | 274(60.9%) | 176(39.1%) |  | 162(62.5%) | 97(37.5%) |  |
|  | Media | 1.13 | 1.18 |  | 1.13 | 1.18 |  | 1.19 | 1.24 |  | 1.17 | 1.20 |  |
|  | IQR | 1.00-1.46 | 1.04-1.70 |  | 1.00-1.44 | 1.03-1.54 |  | 1.09-1.34 | 1.09-1.40 |  | 1.00-1.43 | 1.09-1.44 |  |
|  | Missing | 914(28.3%) | 222(6.9%) |  | 202(25.0%) | 60(7.4%) |  | 10(2.2%) | 9(2.0%) |  | 3(1.2%) | 2(0.8%) |  |
| SG | |  |  | 0.000 |  |  | 0.209 |  |  | 0.747 |  |  | 0.669 |
|  | No. pts (%) | 2409(74.6%) | 821(25.4%) |  | 603(74.6%) | 205(25.4%) |  | 274(60.9%) | 176(39.1%) |  | 162(62.5%) | 97(37.5%) |  |
|  | Media | 1.00 | 1.00 |  | 1.00 | 1.00 |  | 1.00 | 1.00 |  | 1.00 | 1.00 |  |
|  | IQR | 0.99-1.01 | 1.00-1.01 |  | 0.99-1.01 | 1.00-1.01 |  | 1.00-1.00 | 1.00-1.01 |  | 1.00-1.00 | 1.00-1.01 |  |
|  | Missing | 565(17.5%) | 144(4.5%) |  | 138(17.1%) | 35(4.3%) |  | 23(5.1%) | 11(2.4%) |  | 20(7.7%) | 7(2.7%) |  |
| Tbil | |  |  | 0.010 |  |  | 0.578 |  |  | 0.542 |  |  | 0.015 |
|  | No. pts (%) | 2409(74.6%) | 821(25.4%) |  | 603(74.6%) | 205(25.4%) |  | 274(60.9%) | 176(39.1%) |  | 162(62.5%) | 97(37.5%) |  |
|  | Media | 1.26 | 1.36 |  | 1.32 | 1.47 |  | 1.31 | 1.28 |  | 1.09 | 1.33 |  |
|  | IQR | 0.95-1.84 | 1.03-2.28 |  | 0.95-1.99 | 1.10-2.12 |  | 1.00-1.57 | 1.04-1.64 |  | 0.93-1.41 | 1.04-1.57 |  |
|  | Missing | 1067(33.0%) | 260(8.0%) |  | 243(30.1%) | 67(8.3%) |  | 14(3.1%) | 9(2.0%) |  | 81(31.3%) | 14(5.4%) |  |
| DBIL | |  |  | 0.027 |  |  | 0.646 |  |  | 0.052 |  |  | 0.000 |
|  | No. pts (%) | 2409(74.6%) | 821(25.4%) |  | 603(74.6%) | 205(25.4%) |  | 274(60.9%) | 176(39.1%) |  | 162(62.5%) | 97(37.5%) |  |
|  | Media | 1.14 | 1.25 |  | 1.22 | 1.37 |  | 0.89 | 1.01 |  | 0.00 | 1.03 |  |
|  | IQR | 0.80-1.92 | 0.82-2.10 |  | 0.80-1.96 | 0.93-2.20 |  | 0.69-1.28 | 0.80-1.37 |  | 0.00-1.15 | 0.71-1.36 |  |
|  | Missing | 965(29.9%) | 235(7.3%) |  | 219(27.1%) | 61(7.5%) |  | 12(2.7%) | 10(2.2%) |  | 3(1.2%) | 2(0.8%) |  |
| IBIL | |  |  | 0.002 |  |  | 0.213 |  |  | 0.535 |  |  | 0.001 |
|  | No. pts (%) | 2409(74.6%) | 821(25.4%) |  | 603(74.6%) | 205(25.4%) |  | 274(60.9%) | 176(39.1%) |  | 162(62.5%) | 97(37.5%) |  |
|  | Media | 1.26 | 1.40 |  | 1.30 | 1.49 |  | 1.22 | 1.13 |  | 0.87 | 1.19 |  |
|  | IQR | 0.91-1.93 | 0.99-2.30 |  | 0.91-1.99 | 1.03-2.45 |  | 0.94-1.60 | 0.87-1.56 |  | 0.63-1.25 | 0.91-1.50 |  |
|  | Missing | 1097(34.0%) | 285(8.8%) |  | 252(31.2%) | 82(10.1%) |  | 32(7.1%) | 30(6.7%) |  | 3(1.2%) | 7(2.7%) |  |
| TP | |  |  | 0.027 |  |  | 0.653 |  |  | 0.134 |  |  | 0.087 |
|  | No. pts (%) | 2409(74.6%) | 821(25.4%) |  | 603(74.6%) | 205(25.4%) |  | 274(60.9%) | 176(39.1%) |  | 162(62.5%) | 97(37.5%) |  |
|  | Media | 0.97 | 0.98 |  | 0.97 | 0.97 |  | 0.97 | 0.96 |  | 0.95 | 0.97 |  |
|  | IQR | 0.90-1.04 | 0.91-1.08 |  | 0.90-1.05 | 0.89-1.04 |  | 0.92-1.03 | 0.92-1.00 |  | 0.88-1.02 | 0.91-1.03 |  |
|  | Missing | 939(29.1%) | 228(7.1%) |  | 213(26.4%) | 61(7.5%) |  | 11(2.4%) | 9(2.0%) |  | 3(1.2%) | 2(0.8%) |  |
| Alb | |  |  | 0.022 |  |  | 0.694 |  |  | 0.346 |  |  | 0.000 |
|  | No. pts (%) | 2409(74.6%) | 821(25.4%) |  | 603(74.6%) | 205(25.4%) |  | 274(60.9%) | 176(39.1%) |  | 162(62.5%) | 97(37.5%) |  |
|  | Media | 0.96 | 0.97 |  | 0.95 | 0.96 |  | 1.01 | 1.01 |  | 0.89 | 0.99 |  |
|  | IQR | 0.88-1.06 | 0.89-1.08 |  | 0.88-1.05 | 0.89-1.06 |  | 0.94-1.08 | 0.94-1.06 |  | 0.82-0.99 | 0.92-1.06 |  |
|  | Missing | 937(29.0%) | 227(7.0%) |  | 213(26.4%) | 61(7.5%) |  | 11(2.4%) | 9(2.0%) |  | 3(1.2%) | 2(0.8%) |  |
| GGT | |  |  | 0.291 |  |  | 0.482 |  |  | 0.979 |  |  | 0.035 |
|  | No. pts (%) | 2409(74.6%) | 821(25.4%) |  | 603(74.6%) | 205(25.4%) |  | 274(60.9%) | 176(39.1%) |  | 162(62.5%) | 97(37.5%) |  |
|  | Media | 0.94 | 1.02 |  | 0.94 | 0.98 |  | 1.06 | 1.02 |  | 0.77 | 1.15 |  |
|  | IQR | 0.61-1.83 | 0.61-2.38 |  | 0.66-1.71 | 0.64-1.74 |  | 0.81-1.53 | 0.81-1.60 |  | 0.54-1.06 | 0.80-1.49 |  |
|  | Missing | 968(30.0%) | 234(7.2%) |  | 218(27.0%) | 61(7.5%) |  | 11(2.4%) | 9(2.0%) |  | 3(1.2%) | 2(0.8%) |  |
| ALP | |  |  | 0.003 |  |  | 0.960 |  |  | 0.848 |  |  | 0.108 |
|  | No. pts (%) | 2409(74.6%) | 821(25.4%) |  | 603(74.6%) | 205(25.4%) |  | 274(60.9%) | 176(39.1%) |  | 162(62.5%) | 97(37.5%) |  |
|  | Media | 0.88 | 0.87 |  | 0.90 | 0.85 |  | 0.94 | 0.89 |  | 0.85 | 0.88 |  |
|  | IQR | 0.69-1.18 | 0.69-1.21 |  | 0.72-1.18 | 0.69-1.13 |  | 0.73-1.14 | 0.74-1.11 |  | 0.72-1.02 | 0.72-1.09 |  |
|  | Missing | 967(29.9%) | 234(7.2%) |  | 219(27.1%) | 61(7.5%) |  | 11(2.4%) | 9(2.0%) |  | 3(1.2%) | 2(0.8%) |  |
| Na | |  |  | 0.017 |  |  | 0.767 |  |  | 0.001 |  |  | 0.000 |
|  | No. pts (%) | 2409(74.6%) | 821(25.4%) |  | 603(74.6%) | 205(25.4%) |  | 274(60.9%) | 176(39.1%) |  | 162(62.5%) | 97(37.5%) |  |
|  | Media | 1.00 | 1.00 |  | 1.00 | 1.00 |  | 1.00 | 1.00 |  | 0.98 | 1.00 |  |
|  | IQR | 0.99-1.02 | 0.99-1.03 |  | 0.99-1.02 | 0.99-1.02 |  | 0.99-1.01 | 1.00-1.02 |  | 0.97-0.99 | 0.99-1.01 |  |
|  | Missing | 949(29.4%) | 232(7.2%) |  | 214(26.5%) | 60(7.4%) |  | 10(2.2%) | 10(2.2%) |  | 2(0.8%) | 2(0.8%) |  |
| K | |  |  | 0.030 |  |  | 0.818 |  |  | 0.640 |  |  | 0.247 |
|  | No. pts (%) | 2409(74.6%) | 821(25.4%) |  | 603(74.6%) | 205(25.4%) |  | 274(60.9%) | 176(39.1%) |  | 162(62.5%) | 97(37.5%) |  |
|  | Media | 0.93 | 0.93 |  | 0.93 | 0.93 |  | 0.89 | 0.91 |  | 0.87 | 0.89 |  |
|  | IQR | 0.87-1.02 | 0.88-1.04 |  | 0.86-1.00 | 0.87-0.99 |  | 0.87-0.96 | 0.84-0.96 |  | 0.82-0.92 | 0.84-0.93 |  |
|  | Missing | 949(29.4%) | 232(7.2%) |  | 213(26.4%) | 60(7.4%) |  | 10(2.2%) | 10(2.2%) |  | 2(0.8%) | 2(0.8%) |  |
| Cr | |  |  | 0.090 |  |  | 0.374 |  |  | 0.646 |  |  | 0.997 |
|  | No. pts (%) | 2409(74.6%) | 821(25.4%) |  | 603(74.6%) | 205(25.4%) |  | 274(60.9%) | 176(39.1%) |  | 162(62.5%) | 97(37.5%) |  |
|  | Media | 0.99 | 1.03 |  | 1.01 | 0.97 |  | 1.00 | 1.01 |  | 0.92 | 0.97 |  |
|  | IQR | 0.86-1.26 | 0.89-1.39 |  | 0.89-1.22 | 0.84-1.24 |  | 0.89-1.11 | 0.91-1.16 |  | 0.79-1.05 | 0.86-1.10 |  |
|  | Missing | 916(28.4%) | 226(7.0%) |  | 205(25.4%) | 59(7.3%) |  | 10(2.2%) | 9(2.0%) |  | 3(1.2%) | 2(0.8%) |  |
| UA | |  |  | 0.010 |  |  | 0.711 |  |  | 0.172 |  |  | 0.000 |
|  | No. pts (%) | 2409(74.6%) | 821(25.4%) |  | 603(74.6%) | 205(25.4%) |  | 274(60.9%) | 176(39.1%) |  | 162(62.5%) | 97(37.5%) |  |
|  | Media | 1.23 | 1.30 |  | 1.22 | 1.19 |  | 1.26 | 1.30 |  | 1.10 | 1.23 |  |
|  | IQR | 1.01-1.55 | 1.05-1.69 |  | 1.01-1.54 | 1.02-1.61 |  | 1.05-1.52 | 1.12-1.54 |  | 0.92-1.28 | 1.06-1.40 |  |
|  | Missing | 923(28.6%) | 227(7.0%) |  | 207(25.6%) | 59(7.3%) |  | 10(2.2%) | 9(2.0%) |  | 3(1.2%) | 2(0.8%) |  |
| BUN | |  |  | 0.019 |  |  | 0.724 |  |  | 0.017 |  |  | 0.003 |
|  | No. pts (%) | 2409(74.6%) | 821(25.4%) |  | 603(74.6%) | 205(25.4%) |  | 274(60.9%) | 176(39.1%) |  | 162(62.5%) | 97(37.5%) |  |
|  | Media | 1.19 | 1.22 |  | 1.20 | 1.11 |  | 1.28 | 1.38 |  | 1.04 | 1.24 |  |
|  | IQR | 0.92-1.67 | 0.95-1.84 |  | 0.96-1.62 | 0.91-1.52 |  | 1.07-1.47 | 1.11-1.60 |  | 0.84-1.36 | 1.10-1.55 |  |
|  | Missing | 1152(35.7%) | 279(8.6%) |  | 255(31.6%) | 70(8.7%) |  | 10(2.2%) | 9(2.0%) |  | 52(20.1%) | 8(3.1%) |  |
| BMI | |  |  | 0.575 |  |  | 0.401 |  |  | 0.023 |  |  | 0.010 |
|  | No. pts (%) | 2409(74.6%) | 821(25.4%) |  | 603(74.6%) | 205(25.4%) |  | 274(60.9%) | 176(39.1%) |  | 162(62.5%) | 97(37.5%) |  |
|  | Media | 23.77 | 24.22 |  | 23.94 | 24.22 |  | 24.22 | 24.62 |  | 23.41 | 25.13 |  |
|  | IQR | 21.83-25.71 | 22.23-25.95 |  | 22.49-25.65 | 22.14-26.24 |  | 22.04-25.71 | 22.84-26.45 |  | 22.02-25.83 | 23.67-27.03 |  |
|  | Missing | 676(20.9%) | 176(5.4%) |  | 166(20.5%) | 45(5.6%) |  | 0(0.0%) | 0(0.0%) |  | 114(44.0%) | 19(7.3%) |  |
| nsPCa = non-significant prostate cancer; csPCa = Clinically significant prostate cancer; IQR = interquartile range  ^*^ Student't Test.  ^#^ Mann-Whitney U test.  ^§^Chi-square Test. | | | | | | | | | | | | | |
