# Supplementary material for: Artificial intelligence for the diagnosis of clinically significant prostate cancer based on multimodal data: a multicenter study
Source: BMC Med. 2023 Jul 24;21:270. doi: 10.1186/s12916-023-02964-x (PMC10367399; doi:10.1186/s12916-023-02964-x)
Supplement: Supplementary file 3 — Additional file 3: Table S3. Clinical researchcenters involved in this study. [file 12916_2023_2964_MOESM3_ESM.docx]

**Additional file 3: Table S3.** Clinical research centers involved in this study.

| **centers** | **abbreviation** | **start** | **end** | **retrospective** | **prospective** |
| --- | --- | --- | --- | --- | --- |
| Zhongda Hospital Southeast University | Zhongda | 2013/10/23 | 2019/09/11 | 355 | 259 |
| Changhai Hospital of Shanghai | Changhai | 2012/10/22 | 2019/04/09 | 1517 | 450 |
| The First Affiliated Hospital of Soochow University | Suda | 2008/5/13 | 2019/6/18 | 646 | / |
| the First Affiliated Hospital of Xi’an Jiaotong University | XJTU | 2012/1/4 | 2019/11/19 | 667 | / |
| West China Hospital,Sichuan University | Huaxi | 2012/9/24 | 2019/2/20 | 418 | / |
| Shanghai Ninth People’s Hospital | Shanghai_9H | 2021/1/1 | 2021/10/30 | 46 | / |
| The Second Attached Hospital Of Fujian Medical University | Fujian_2H | 2016/1/1 | 2021/12/31 | 103 | / |
| The first people of Yulin | Yulin_1H | 2017/10/25 | 2021/12/13 | 72 | / |
| Jinling Hospital, Nanjing University | Jinling | 2016/1/1 | 2021/11/30 | 214 | / |
