# Supplementary figures and images for: Artificial intelligence for the diagnosis of clinically significant prostate cancer based on multimodal data: a multicenter study
Source: BMC Med. 2023 Jul 24;21:270. doi: 10.1186/s12916-023-02964-x (PMC10367399; doi:10.1186/s12916-023-02964-x)

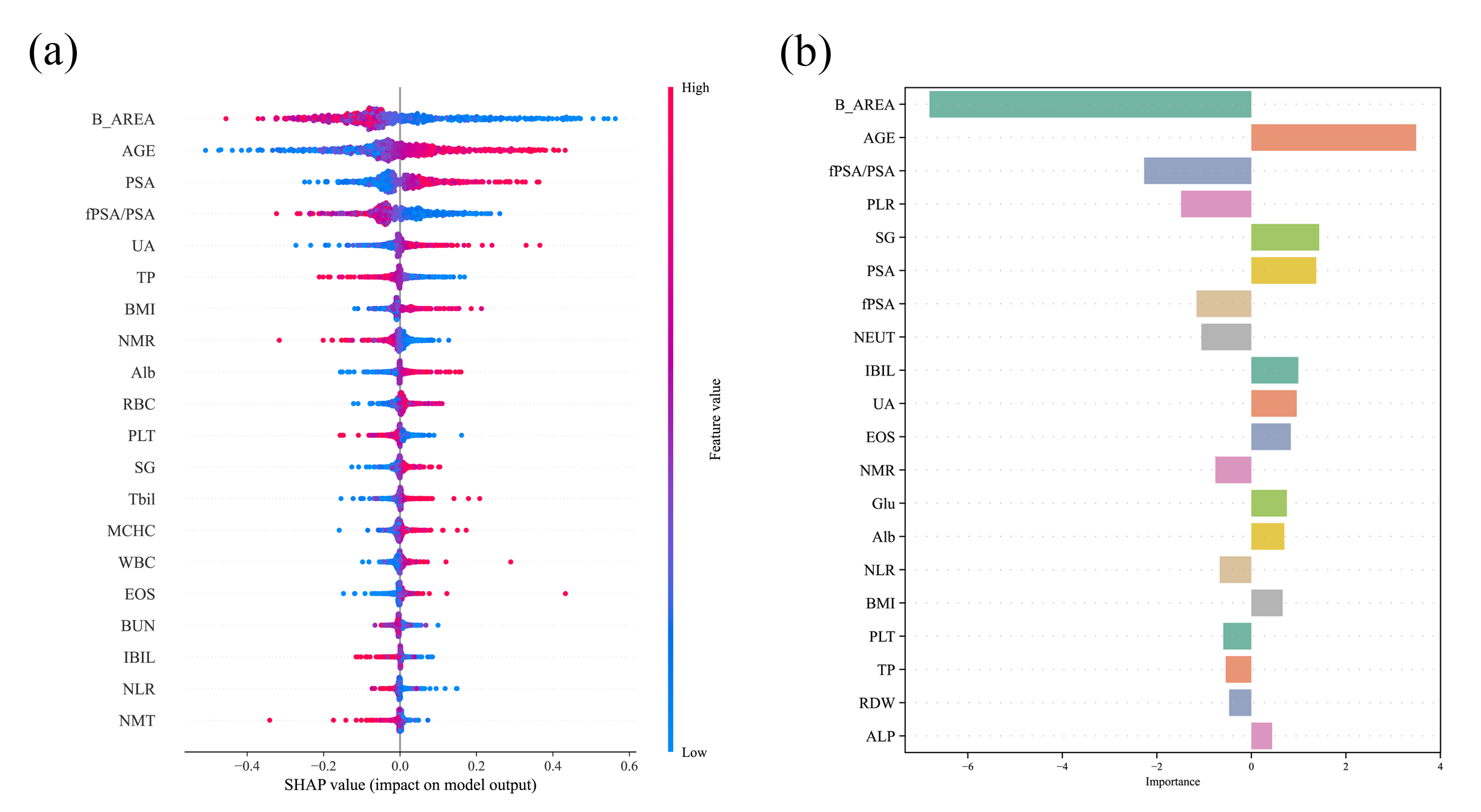

Supplement: Supplementary file 4 — Additional file 4: Figure S1. Featureimportance analysis uses AutoML. (a) SHAP summary plot. SHAP feature importancemeasured as the mean absolute Shapley values. (b). [file 12916_2023_2964_MOESM4_ESM.tif]
